# Sternal Nonunion: A Novel Approach to Reconstruction

**Published:** 2012-08-02

**Authors:** Nicola A. Quatrano, Michael M. Van Vliet, Emily B. Ridgway

**Affiliations:** ^a^Geisel School of Medicine at Dartmouth, Hanover, NH; ^b^Department of Plastic and Reconstructive Surgery, Dartmouth-Hitchcock Medical Center, Lebanon, NH

## DESCRIPTION

**Figure 1:**
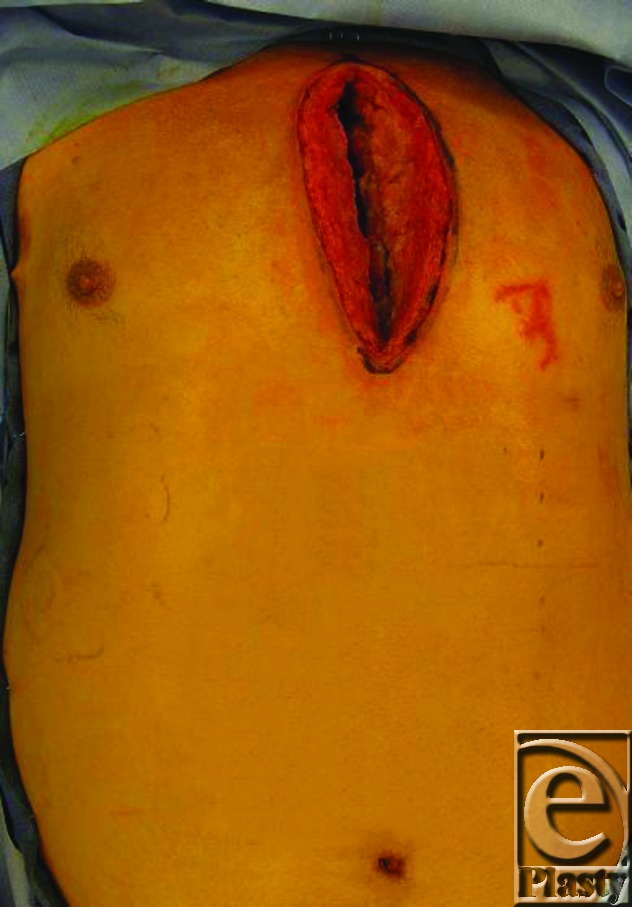
Noninfected sternal nonun-ion after coronary artery bypass graft surgery.

A 54-year-old man with multiple comorbidities including wheelchair use secondary to a left below knee amputation, underwent a 2-vessel coronary artery bypass graft surgery and subsequently developed a noninfected sternal nonunion (Fig 1). Subsequent sternal reconstruction was necessary and entailed an innovative approach given the patient's need for pectoralis major muscle function for transfer in and out of wheel chairs.

## QUESTIONS

1. **What is the current recommendation for coverage of mediastinal wounds?**
2. **When are local muscle flaps indicated, and when are they not indicated?**
3. **What other options are there besides local muscle?**

## DISCUSSION

Myocutaneous flaps are the preferred time-honored approach for sternotomy wound closure following sternal nonunion and mediastinitis.^1^ Limitations of these flaps include the morbidities of functional impairment directly associated with loss of muscle and long operative and anesthetic times in a typically high-risk patient population secondary to multiple comorbidities.^2^^,^^3^ With this in mind, our institution considers the deep superior epigastric artery perforator flap to serve as an alternative option in sternal reconstruction.

Perforators of the deep superior epigastric artery (DSEA) have been the basis for both myocutaneous and fasciocutaneous flaps applied in a variety of clinical settings in the past.^3^ In 2009, Mah et al^3^ shared a 3-case series in which transversely oriented pedicled fasciocutaneous flaps based on DSEA perforators were used for sternal reconstruction. Despite efforts to design the flaps according to vascular territories, 2 of the 3 patients experienced tip necrosis. To address this major complication, our institution suggests an improvement in flap design through vertical orientation based on the previously delineated angiosome of the DSEA.^4^ Patterns of anastomosis between the DSEA and deep inferior epigastric artery (DIEA) have been described in radiographic cadaveric studies as anatomic territories bounded by choke vessels which unite the 2 systems in the segment of muscle above the umbilicus.^4^ Vertical flap orientation provides adequate length to cover large sternal defects while providing reliable vascular supply from the DSEA-DIEA axis.

Sternal wound dehiscence presents a reconstructive challenge especially in clinical situations where muscle loss becomes unfeasible. Deep superior epigastric artery-based fasciocutaneous flaps offer an alternative with several advantages including the preservation of rectus abdominis or pectoralis major muscles for future myocutaneous flaps, prevention of functional impairment associated with loss of muscle, and the benefit of shorter operative and anesthetic times in a patient population with typically high surgical risks.^2^^,^^3^ Our institution makes a more specific recommendation in the design of this flap to address the major complication of tip necrosis seen with the transverse orientation. On the basis of the previously delineated angiosome of the DSEA, we offer an improvement in flap design by endorsing a vertical orientation, which affords greater vertical coverage for sternal wounds and reliable vascular supply.^4^

**Figure 2:**
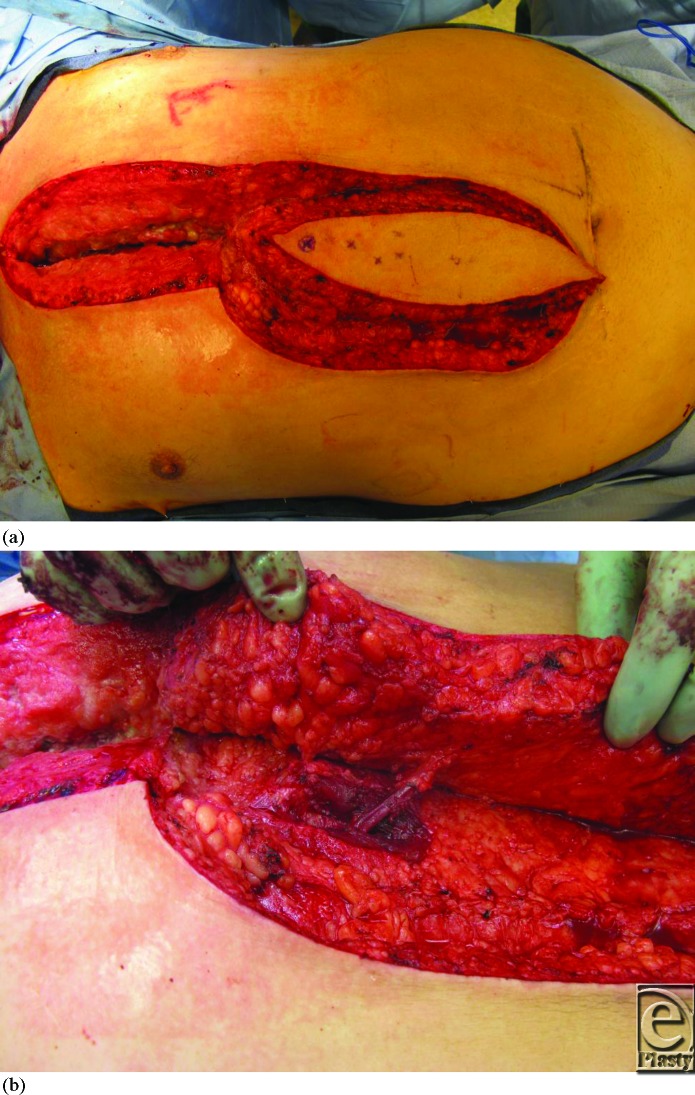
Intraoperative picture of flap design (*a*) and skeletonized single perforating vessel from the deep superior epigastric artery (*b*).

**Figure 3:**
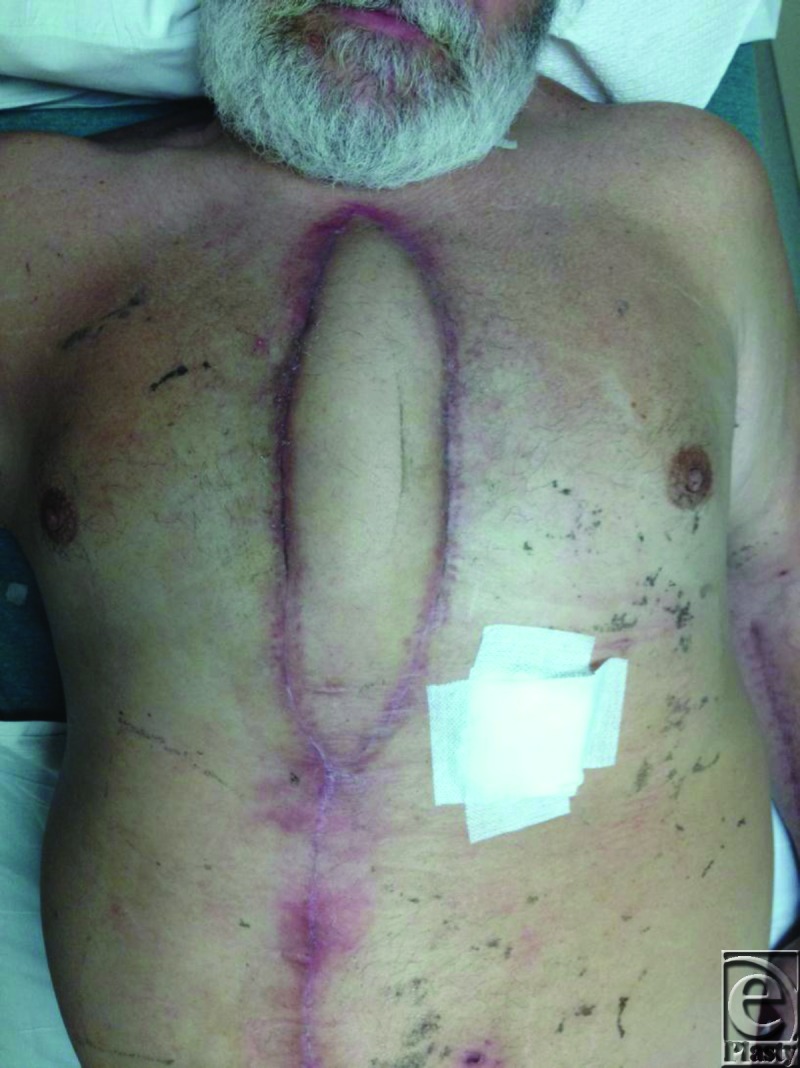
Healed flap with excellent viability at 6-week postsurgery.

For this patient, because pectoralis major muscle function is essential for transfer in and out of wheel chairs, the possibility of functional impairment associated with muscle loss was unacceptable and an alternative reconstructive option was pursued. A fasciocutaneous flap based on a single DSEA perforator was designed as a vertical ellipse and rotated 180 degrees clockwise after skeletonizing the perforator to cover the defect (Fig 2). The length of the operation was 117 minutes. At 6 weeks after surgery, the flap was viable without evidence of tip necrosis (Fig 3).
